# Sphingomyelin in Brain and Cognitive Development: Preliminary Data

**DOI:** 10.1523/ENEURO.0421-18.2019

**Published:** 2019-08-05

**Authors:** Nora Schneider, Jonas Hauser, Manuel Oliveira, Elise Cazaubon, Sara Colombo Mottaz, Barry V. O’Neill, Pascal Steiner, Sean C. L. Deoni

**Affiliations:** 1 Société des Produits Nestlé SA, Nestlé Research, 1000 Lausanne, Switzerland; 2 Department of Pediatrics, Brown University, Providence, RI 02912; 3 Memorial Hospital of Rhode Island, Pawtucket, RI 02860

**Keywords:** brain development, cognitive development, infants, myelination, oligodendrocytes, sphingomyelin

## Abstract

Sphingomyelin (SM) supports brain myelination, a process closely associated with cognitive maturation. The presence of SM in breast milk suggests a role in infant nutrition; however, little is known about SM contribution to healthy cognitive development. We investigated the link between early life dietary SM, later cognitive development and myelination using an exploratory observational study of neurotypical children. SM levels were quantified in infant nutrition products fed in the first three months of life and associated with myelin content (brain MRI) as well as cognitive development (Mullen scales of early learning; MSEL). Higher levels of SM were significantly associated with higher rates of change in verbal development in the first two years of life (*r* = 0.65, *p* < 0.001), as well as, higher levels of myelin content at 12–24 months, delayed onset and/or more prolonged rates of myelination in different brain areas. Second, we explored mechanisms of action using in vitro models (Sprague Dawley rat pups). In vitro data showed SM treatment resulted in increased proliferation [*p* = 0.0133 and *p* = 0.0434 at 4 and 10 d *in vitro* (DIV)], maturation (*p* = 0.467 at 4 d DIV) and differentiation (*p* = 0.0123 and *p* = 0.0369 at 4 and 10 DIV) of oligodendrocyte precursor cells (OPCs), as well as increased axon myelination (*p* = 0.0005 at 32 DIV). These findings indicate an impact of dietary SM on cognitive development in healthy children, potentially modulated by oligodendrocytes and increased axon myelination. Future research should include randomized controlled trials to substantiate efficacy of SM for cognitive benefits together with preclinical studies examining SM bioavailability and brain uptake.

## Significance Statement

The presences of sphingomyelin (SM) in breast milk suggests it has an important role in infant nutrition. Ways of measuring SM in milk are only recently developed, meaning little is known about its role in brain development. In this study, we used two approaches to explore this further. First, we investigated the link between early life SM obtained from the diet with later brain development and brain structure in a group of healthy children. Second, we examined the mechanism behind these effects using experimental models of brain development. Our findings in children indicate a positive impact of SM on brain development supported by experimental models. Future studies need to investigate further to build on the findings of our preliminary work.

## Introduction

Sphingomyelin (SM) is the most abundant eukaryotic sphingolipid and one of the major constituents of cell membranes (Gault et al., 2010). It is enriched in the CNS (Babin et al., 1993) and particularly abundant in the myelin sheath that surrounds neuronal axons (Pierre, 1984). SM has been reported to play an important role in cell processes (Merrill and Sandhoff, 2002; Hirabayashi and Furuya, 2008), the regulation of inflammatory responses (Assi et al., 2013; Maceyka and Spiegel, 2014), and signal transduction (Lingwood and Simons, 2010). Moreover, due to its critical role in myelin integrity and function (Don et al., 2014) as well as in axonal maturation (Ledesma et al., 1999), SM may be a relevant lipid during brain development. In particular, SM may play a prominent role from mid-gestation to the end of the first postnatal year when CNS myelin dramatically increases (Tau and Peterson, 2010). Ensuring efficient transmission of nerve impulses along neuronal axons (Fields, 2008; Stiles and Jernigan, 2010), CNS myelination is important in the maturation of brain networks, coordinated information processing and ultimately cognitive performance in infants and children (O’Muircheartaigh et al., 2013, 2014; Deoni et al., 2014). Therefore, SM may play an important role in cognitive development via its structural and functional involvement in CNS myelination.

SM is present in human breast milk and is the major phospholipid in human milk fat accounting for ∼37% of the phospholipid fraction (Bitman et al., 1984; Oshida et al., 2003). Mature breast milk contains SM at levels between 31 and 153 mg/l (Cilla et al., 2016), indicating high variability. However, despite these levels in breast milk, the functional role of dietary SM on cognitive development or its physiologic impact on underlying neurodevelopmental processes like myelin formation, remains poorly understood. In fact, only one dietary intervention study has evaluated the effect of SM supplementation on cognition in human infants. In a sample of low-birth weight infants, the feeding of SM-fortified infant formula (20% vs 13% total milk phospholipids) in the first eight weeks of life resulted in increased plasma and erythrocyte SM levels at four, six, and eight weeks of life. In addition, the same study reported improved behavior rating scores (behavior rating scale of the Bayley scales of infant and toddler development; BSID-II), better novelty preference scores (Fagan test), lower latency of visual evoked potentials and increased sustained attention scores (free-play sustained attention test of Colombo) at 12 and/or 18 months of age; no differences were found for neurodevelopment scores (BSID-II; Tanaka et al., 2013). The underlying mechanism for those findings were not investigated, but improvements in myelination (potentially influenced by SM) were speculated. In another randomized controlled trial in healthy infants a low-energy, low-protein formula supplemented with bovine milk fat globule membranes, containing SM, was compared to a standard formula and to a reference group of breastfed infants. The intervention (two to six months of life) resulted in higher cognitive scores at 12 months of age in the experimental group versus control group. It also resulted in similar cognitive scores when comparing the experimental group to the breastfed infants (Timby et al., 2014). These studies suggest SM is a relevant nutritional contributor to brain and cognitive development.

Alongside the aforementioned clinical investigations, previous rodent studies provide support for SM in brain and cognitive development. Specifically, a SM-enriched diet positively affects CNS myelination and myelin thickness in experimental conditions of low serine palmitoyltransferase (SPT) activity, a key enzyme for the biosynthesis of sphingosine and a precursor of many sphingolipids, including SM (Oshida et al., 2003). With regard to cognitive performance, early life rats supplemented with a 1% complex milk lipid, which includes SM, resulted in an improved novelty memory performance and an improvement in learning of a spatial memory task in adulthood (Vickers et al., 2009).

In summary, the enriched presence of SM in the CNS and maternal breast milk, alongside its involvement in myelination suggest a prominent role in early life brain and cognitive development. However, the impact of dietary intake of SM as well as underlying mechanisms of action are mostly unknown, especially in healthy infants and young children. We investigated the link between early life dietary SM, developmental myelination and later cognitive development in a cohort of neurotypical infants and young children. Furthermore, we investigated potential mechanisms for SM-related effects on myelination using *in vitro* models.

## Materials and Methods

### Observational cohort study

Infants and children recruited for this study were recruited from the Brown University Assessment of Myelination and Behavior Across Maturation (BAMBAM) as part of an accelerated-longitudinal study focused on characterizing normal brain development. Specific exclusion criteria included *in utero* alcohol or illicit substance exposure, premature (<37 weeks of gestation) or multiple birth, fetal ultrasound abnormalities, complicated pregnancy (e.g., preeclampsia), Activity, Pulse, Grimace, Appearance, Respiration (APGAR) scores <8, neonatal intensive care unit admission, neurologic disorder (e.g., head injury, epilepsy), psychiatric or developmental disorders in the infant, parents or siblings (including maternal depression requiring medication). Eligibility was determined via a pre-enrolment interview and verified by family and medical history questionnaire updates throughout the study. To date, >450 typically developing infants and toddlers have been recruited and assessed at regular intervals through infancy and childhood. Infants (*N* = 236, 132 male), defined as children under two years of age, have received biannual MRI and neurocognitive assessments from age at enrolment until two years of age, with annual assessments thereafter. Toddlers (*N* = 218, 122 male), defined as children older than two years, have received annual MRI and neurocognitive assessments from age of enrolment. Though an accelerated-longitudinal study design, some children have to-date received only one scan and assessment. Thus, there is a combination of cross-sectional and longitudinal data available. This study was conducted according to the guidelines laid down in the Declaration of Helsinki and all procedures involving human subjects were approved by the host institution ethical committee [Brown University, 0911000083]. Written informed consent was obtained from all subjects.

For our exploratory and retrospective observational study on dietary SM, brain and cognitive development, we selected all children from the larger study cohort with data on their early life feeding (i.e., exclusive infant nutrition product feeding). This resulted in *N* = 150 infants and toddlers that could be considered for the specific analyses on the influence of SM intake on brain myelination and cognitive development. This group was further refined by including only those who could be grouped into the biggest homogenous infant nutrition product groups as available from the cohort, see further details in the next section.

### Nutritional intake and infant nutrition product composition analysis

Infant nutrition product intake was provided by parents as part of child history information. For our study, only children receiving the same product for at least 80% of feeds during the first three months (90 d) of life were included. Three products were found to be predominant (*N* = 88), with 44% of children fed product A, 32% fed product B, and 24% fed product C. Quantification of SM in products A, B and C was determined retrospectively at Neotron analytical laboratories (Italy) using the method of Giuffrida et al. (2013). This validated method has a SM quantification limit of <200 mg/kg. The products for analyses were obtained from commercial providers (local supermarket in the Rhode Island area) at the study end, not during the actual feeding period of the infants. Demographic information for each group is provided in Table 1. Groups were, in general, matched for gender composition, parent marital status, birth weight, maternal education (as denoted by the education scale of the Hollingshead Four-factor Index of socioeconomic status). There was a significant group difference in gestational age between the Product A (∼40 weeks) and product B children (∼39.3 weeks); however, both groups are considered full term.

**Table 1.** Demographic information

		All infants		Product A		Product B		Product C		ANOVA *p*	χ^2^ *p*
Gender	Male (*n*)	54		24		18		12			0.88
	Female (*n*)	34		15		10		9			
Marital status	Married/living together (*n*)	68		29		21		18			0.57
	Divorced/single (*n*)	20		10		7		3			
Age range (d)		88–3409		88–3384		98–3308		103–3409			
		Mean	SD	Mean	SD	Mean	SD	Mean	SD		
Gestation (d)		281	19	285	12	275	11	278	6	A vs B, *p* < 0.01	
Birth weight (g)		3313	540	3446	508	3179	555	3164	589	0.07	
Maternal education(y)		5.6	1.5	5.6	1.8	5.4	1.1	5.8	1.2	0.64	
Number of scans per child		2.5	1.3	2.1	1.3	3.0	1.2	2.2	1.4	A vs B, *p* = 0.01	
Mean inter-scan interval (d)		380	250	330	190	407	299	439	250	0.21	

### Cognitive development

To assess emerging cognitive abilities, all children underwent a neuropsychological assessment by trained and qualified medical and psychological staff within 7 d of a successful MRI. For the age group included herein, the cognitive outcome measure was the Mullen scales of early learning (MSEL; Mullen, 1995). The MSEL is a standardized and population-normed tool for measuring early cognitive abilities in children under 68 months of age through tasks of visual reception, expressive and receptive language, fine and gross motor function. In this study, we considered the verbal developmental quotient (VDQ), based on the receptive and expressive language subscales, the non-VDQ (NVDQ) based on the visual reception and fine motor subscales, as well as the early learning composite (ELC) based on the subscales visual reception, fine motor, expressive language, and receptive language. The composite scores can be calculated with a population mean of 100 and a SD of 15.

### MR imaging

MRI scans from infants were acquired during natural non-sedated sleep in a 3T Siemens Tim Trio scanner. To minimize motion, children were swaddled with a pediatric MedVac vacuum immobilization bag (CFI Medical Solutions) and secured with foam cushions. Scanner noise was reduced by reducing the imaging speed and using a noise-insulating scanner bore insert (Quiet Barrier HD Composite, Ultra Barrier). Pediatric ear covers and electrodynamic headphones (MR Confon) were also used (Dean et al., 2014a). For MRI sequences please refer to Table 2.

**Table 2.** MRI sequences and parameters

MRI sequence	Sequence parameters	Time
mcDESPOT	Age-group protocols drawn from Dean et al. (2014b)	18–25:00
T1W MP-RAGE	(1.2 × 1.2 × 1.2) mm^3^, TR = 1800 ms, TE = 2.2 ms, TI = 900 ms, flip angle = 15°, FOV = 220 × 220 mm^2^, 134 slices, GRAPPA = 2	4:00

MC DESPOT = multicomponent-driven equilibrium single pulse observation of T1 and T2; MP-RAGE = magnetization-prepared rapid gradient-echo; DTI = diffusion-tensor imaging; rsMRI = resting state MRI; TR = repetition time; TE = echo time; TI = inversion time; FOV = field of view.

To assess brain myelin content and growth, neuroimaging with multicomponent-driven equilibrium single pulse observation of T1 and T2 (mcDESPOT; Deoni et al., 2008) was used to quantify the myelin water fraction (MWF), a surrogate measure of myelin volume, throughout the brain. Infants were scanned using optimized protocols described previously (Deoni et al., 2012) and detailed in Table 3. Total imaging times ranged from 18 to 25 min, depending on the age of the child.

**Table 3.** Details of the mcDESPOT acquisition protocol per age-group^(25)^

Age group (months)	3–9	9–16	16–28	28–48
Acquisition time (min:s)	18:22	18:42	21:38	24:20
Field of view (cm^3^)	14 × 14 × 13	17 × 17 × 14.4	18 × 18 × 15	20 × 20 × 15
Unprotected dBa	54	62	69	74
SPGR TR/TE (ms)	12/5.8	12/5.9	12/5.4	11/5.2
SPGR flip angles (°)	2, 3, 4, 5, 7, 9, 11, 14	2, 3, 4, 5, 7, 9, 11, 14	2, 3, 4, 5, 7, 9, 11, 14	2, 3, 4, 5, 7, 9, 12, 16
IR-SPGR TI (ms)	600/950	600/900	500/850	500/800
bSSFP TR/TE (ms)	10/5	10.2/5.1	10/5	9.8/4.4
bSSFP flip angles (°)	9, 14, 20, 27, 34, 41, 56, 70	9, 14, 20, 27, 34, 41, 56, 70	9, 14, 20, 27, 34, 41, 56, 70	9, 14, 20, 27, 34, 41, 56, 70

SPGR = Spoiled gradient-echo; IR-SPGR = inversion recovery SPGR; TR = repetition time; TE = echo time; TI = inversion time; SSFP = steady-state free precession.

Following data collection and standard data preprocessing (Deoni 2011), voxel-wise MWF maps were calculated (Deoni and Kolind, 2015) for each child and then non-linearly aligned to approximate Montreal Neurologic Institute (MNI) space (Avants et al., 2011; Deoni et al., 2012). White matter (WM) masks of five bilateral regions (frontal, temporal, occipital, parietal, and cerebellar WM) as well as the body, genu, and splenium of the corpus, were created from common WM atlases and superimposed onto each child’s MWF map to derive mean MWF values for each region for each child.

### SM, brain myelin, and cognition

For statistical analyses and a better understanding of developmental changes with age, we considered the following three groups based on age, and performed both cross-sectional and longitudinal assessments: (1) cross-sectional analysis of children between 1 and 12 months of age (*N* = 39, 17 male); 2) cross-sectional analysis of children between 12 and 24 months of age (*N* = 21, 8 male); 3) longitudinal analysis of children between one month and six years of age (*N* = 88, 54 male).

#### Cross-sectional analysis

For groups 1 and 2, the correlation between MWF and product SM content was evaluated at each image voxel throughout the brain using a simple linear correlation approach that accounted for child age. A general linear model (GLM) was constructed that modeled MWF as the outcome variable and product SM content and child age at time of scan as predictor variables. Analysis was performed at each image voxel with significance defined as *p* < 0.05 corrected for multiple comparisons using a threshold-free cluster-based technique (Smith and Nichols, 2009).

To compare measures of cognitive ability, an unpaired *t* test was used to test the difference in ELC, VDQ, and NVDQ between children fed product A and product B. The product C group was omitted from this group analysis due to its small size in each age group. It was included in all other analyses. Significance was defined as *p* < 0.017 (*p* < 0.05 corrected for the three analyses using Bonferroni correction).

#### Longitudinal analysis

Non-linear mixed-effects modeling was used to fit a modified Gompertz growth model (Dean et al., 2014b) to each child’s longitudinally-acquired MWF data in each of the 13 WM regions of interest. The Gompertz curve is a sigmoidal function defined by four parameters: the initial onset of growth (β), initial growth rate (γ), a second reflection where growth begins to slow considerably (α), and a final linear growth rate (δ). Following estimation of these parameters for each individual, the correlation between each parameter and product SM content was calculated. Statistical significance was defined as *p* < 0.001 (*p* < 0.05 corrected for the 52 tests).

Correlations were also investigated between product SM content and cognitive development trends in a similar manner. Linear mixed-effects modeling was first used to fit a linear model to each child’s repeated ELC, VDQ, and NVDQ data. The correlation between SM content and the slope of the linear model was then calculated, with statistical significance defined as *p* < 0.017 (*p* < 0.05 corrected for the three analyses using Bonferroni correction).

### Preclinical materials and methods

#### Cell treatments

*In vitro* studies were performed using either pure oligodendrocyte precursor cell (OPC) cultures or co-cultures of OPC and primary neurons. Pure OPC and co-cultures were treated with either olesoxime at 0.3 µM, SM (liposomes composed of 75% 1,2-dipalmitoyl-sn-glycero-3-phosphocholine and 25% bovine brain SM, Avanti Polar Lipids) at 0.5, 5, and 25 µM or vehicle (1,2-dipalmitoyl-sn-glycero-3-phosphocholine liposomes). SM doses were selected to represent human CSF levels (Mandal et al., 2012) or higher. Olesoxime was used as positive control (Magalon et al., 2012). For pure OPC and coculture tests both were run in triplicates, for each replication the average of at least two technical replicates per condition per time point was calculated.

##### Pure OPC cultures

Cells were imaged at 4, 7, and 10 d *in vitro* (DIV). OPC proliferation, differentiation and maturation was determined by the total number of 4′,6-diamidino-2-phenylindole (DAPI)+, O4+, and myelin basic protein (MBP)+ cells, respectively.

##### Co-cultures

Ten DIV OPC were plated with 14 DIV neurons. Co-cultures were fixed at 18, 26, and 32 DIV and stained for MBP and β-III-tubulin (marker of axonal growth); co-expression was used as an index of myelination.

For both pure OPC and co-cultures, the nutrient treatment started at 3 DIV (for coculture the DIV count started the day of the neuron-OPC coculture) and was administered via medium change every second day.

#### OPC culture

Cells from Sprague Dawley rat pup (P2) cortices were isolated using papain solution at 37°C for 20 min, processed through a 100-μm gasket and plated at 30,000 cells/cm^2^ in neuronal medium (with 1% FBS) for 3 d. On day 9, a 45-min shaking (50 rpm) in 5% CO_2_ was used to isolate OPC that were then kept for 3 h on 4 ml of glial culture media (DMEM added with FBS 10%, 33 U/ml penicillin and 33 μg/ml streptomycin, GlutaMAX 1%) supplemented with 5 μg/ml insulin. Flask were shaken 16 h at 220 rpm and OPC were isolated by pre-plating on a Petri dish for 30 min, after which supernatant (containing the OPC) was collected. For OPC maturation, OPC were seeded at 30,000 cells/cm^2^.

For OPC-neuron coculture myelination, collected OPC were seeded at 30,000 cells/cm^2^ on hippocampal neurons in a final volume of 1 ml oligodendrocyte media [OL media; DMEM added with 100× OL-Supplement, bovine insulin (from 1 mg/ml stock), GlutaMAX, Holo-transferrin (from 33 mg/ml stock), B27 supplement, FBS, with CNTF (from 50 ng/μl stock) added on subsequent medium changes].

#### Primary neuronal cultures

Hippocampus (E18 Sprague Dawley embryo) was incubated with 2.5% trypsin for 15 min at 37°C, then gently washed and kept in culturing media. Following mechanical dissociation, cells were plated at 50,000/cm^2^ in neuronal plating medium and after 4 h put in compete neuronal culturing medium.

#### Immunofluorescence quantification

Images were taken with ArrayScan XTI HCA Reader (Thermo Fisher Scientific) equipped with a Photometrics X1, 14 bit, high-resolution camera. A Zeiss Plan-NEOFLUAR 20×/0.4 objective was used to capture 20 images per well (resolution of 1104 × 1104 pixels). Florescence was detected with the following settings: excitation LED 386_23 (DAPI), 549_15 (MBP) and 458_20 nm (O4 and β-III-tubulin), penta-band BGRFRN dichroic mirror, BGRFRN emission filter, exposure time of 0.0263 s (DAPI), 0.0553 s (MBP), and 0.266 s (O4) for pure OPC and 0.0233 s (DAPI), 0.1203 s (MBP), and 0.045 s (β-III-tubulin) for co-cultures, respectively. Image analysis was done with HCS Studio software (Thermo Fisher Scientific). MBP and β-III-tubulin colocalization fluorescence was quantified using β-III-tubulin area signal as a mask to determine regions of interest, then measuring the MBP signal in this region (threshold: signal 4× higher than the MBP background signal).

### Data analysis

The effect of different doses of SM on the independent outcomes was assessed using a linear mixed model with treatment as fixed effect and the round as random effect. The effect size was represented by mean difference between groups, estimated by restricted maximum likelihood approach (REML). Data were a-priori divided by the average of the untreated group for each time point. Applying the Benjamini–Hochberg procedure to control the false positive discovery rate at 5% (on 84 performed tests), the 38 *p* <0.022 can all be considered significant (Benjamini and Hochberg, 1995). All the results were expressed as mean (SEM) estimated by the model and the differences are considered to be statistically significant at *p* < 0.05. Statistical analyses were performed using R.3.2.1.

## Results

For statistical tables of each analyses, please see Table 6 and 7.

### Observational cohort study

#### Nutritional composition analyses

SM levels ranged from 28 to 71 mg/l in the three analyzed infant nutrition products, with 28 mg/l in product A, 71 mg/l in product B, 28 mg/l in product C.

#### Cross-sectional results

For an overview of the mean cognitive development scores, please refer to Table 4.

**Table 4.** Mean cognitive scores per infant age and product group

MSEL scores	1–12 months M (SD)				12–24 months M (SD)			
	All products	Product A	Product B	Product C	All products	Product A	Product B	Product C
ELC	93.2 (14.4)	91.5 (15.7)	94.7 (13.8)	94.3 (15.2)	97.1 (13.6)	92.3 (12.4)	104.2 (8.5)	101.8 (20.3)
VDQ	108.1 (21.8)	109.9 (21.4)	105.1 (18.1)	107.2 (19.3)	104.3 (15.6)	96.9 (12.1)	109.5 (18.7)	107 (18.8)
NVDQ	90.8 (21.6)	91.1 (23.8)	90.4 (18.4)	90.7 (16.3)	101 (17.8)	98.8 (14.7)	108.2 (8.4)	95 (13.2)

**Figure 1.**
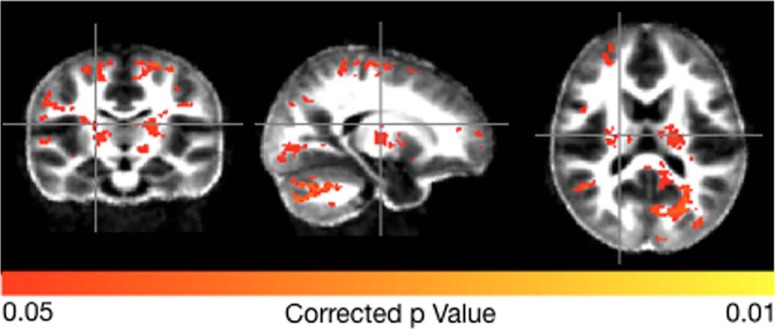
Brain regions with a significant relationship between product SM content and brain MWF in children 12–24 months of age. Colored brain regions are those where associations between SM content and MWF content reached statistical significance (*p* < 0.05 FDR).

From the cross-sectional data spanning 1–12 months, we found no significant group difference in ELC (*p* = 0.56), NVDQ (*p* = 0.56), or VDQ (*p* = 0.94). For the 12- to 24-month data, significant differences before correction were found for ELC (*p* = 0.02) and a trend toward significance for NVDQ (*p* = 0.09) and VDQ (*p* = 0.06), however, these did not remain statistically significant after correction for multiple comparisons. With respect to myelin content, no significant correlations between MWF and product SM content was observed in the 1- to 12-month data; however, significant associations, corrected for multiple comparisons, were found in the 12- to 24-month data (Fig. 1) in bilateral cerebellum (*r* = 0.56, *p* = 0.001), occipital lobe (*r* = 0.48, *p* = 0.03), visual cortex (*r* = 0.44, *p* = 0.04), internal capsule (*r* = 0.58, *p* = 0.001), and parietal lobe and motor cortices (*r* = 0.52, *p* = 0.02).

#### Longitudinal results

**Figure 2.**
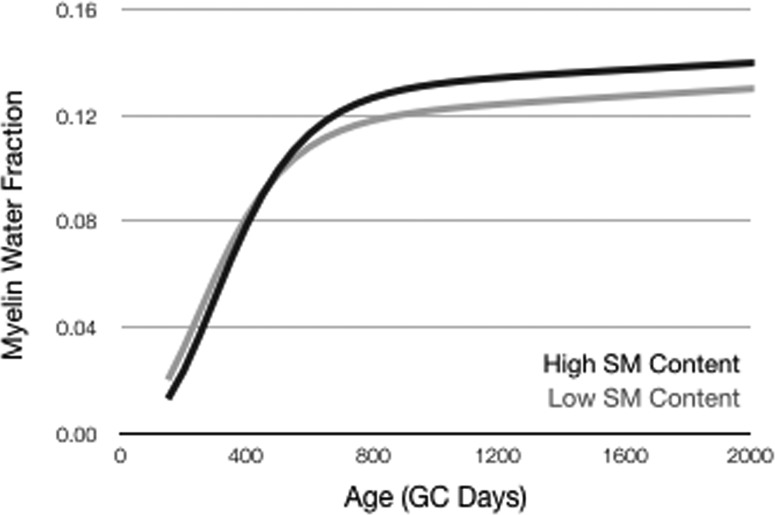
Mean whole-brain WM myelination trajectories derived from children who either received a product composition with high SM content (71 mg/l) or a lower SM content (28 mg/l) composition.

With respect to cognitive development, SM content was significantly and positively associated with the rate of change in VDQ (Pearson’s *r* = 0.65, *p* < 0.001). No significant associations were found for change in NVDQ (Pearson’s *r* = 0.09, *p* = 0.52) and overall ELC following multiple testing correction (Pearson’s *r* = 0.28, *p* = 0.06). A summary of the longitudinal MWF growth for different brain regions and SM content analysis is shown in Table 5. Overall, we note that early life SM content is significantly related to brain myelin development profiles throughout the brain (Fig. 2; Table 5) and, in particular with respect to the initial onset of myelination (Gompertz curve parameter β), i.e., greater SM content being associated with a later myelin onset, and initial growth rate (Gompertz curve parameter γ), i.e., greater SM content being associated with a slower but more prolonged rate of myelination.

**Table 5.** Correlation analysis of each Gompertz growth model and SM content

Region	Parameter	Pearson’s *r*	*p* value
Frontal WM	α	0.08	0.580
	β	–0.51	<0.001***
	γ	–0.21	0.147
	δ	–0.22	0.128
Temporal WM	α	0.04	0.790
	β	–0.99	<0.001***
	γ	–0.83	<0.001***
	δ	0.29	0.040*
Parietal WM	α	0.37	0.008**
	β	–0.90	<0.001***
	γ	–0.99	<0.001***
	δ	–0.97	<0.001***
Occipital WM	α	0.13	0.170
	β	–0.99	<0.001***
	γ	–0.45	0.001***
	δ	0.36	0.012*
Cerebellar WM	α	0.99	<0.001***
	β	–0.68	<0.001***
	γ	–0.83	<0.001***
	δ	0.31	0.026*
Corpus callosum (body)	α	0.37	0.009**
	β	–0.94	<0.001***
	γ	–0.99	<0.001***
	δ	–0.99	<0.001***
Corpus callosum (genu)	α	0.17	0.240
	β	–0.73	<0.001***
	γ	–0.43	0.002**
	δ	–0.74	<0.001***
Corpus callosum (splenium)	α	0.52	0.001***
	β	–0.85	<0.001***
	γ	–0.99	<0.001***
	δ	–0.99	<0.001***

The four Gompertz curve parameters indicate the initial onset of growth (β), initial growth rate (γ), a second reflection where growth begins to slow considerably (α), and a final linear growth rate (δ); *p < 0.05, **p < 0.01, ***p < 0.001.

**Table 6.** Statistical table for observational cohort analyses

	Data structure	Type of test	Power
Cross-sectional group difference in ELC (12–24 months of age)	Normal distribution	*t* test	0.72
Association between MWF and SM content (12–24 months)	Normal distribution	Spearman’s rank-order	0.56
Association between SM content and VDQ development	Normal distribution	Pearson’s *r*	0.5
Association between SM content and temporal WM β	Normal distribution	Pearson’s *r*	>0.90
Association between SM content and temporal WM γ	Normal distribution	Pearson’s *r*	>0.90
Association between SM content and parietal WM β	Normal distribution	Pearson’s *r*	>0.90
Association between SM content and parietal WM γ	Normal distribution	Pearson’s *r*	>0.90
Association between SM content and parietal WM δ	Normal distribution	Pearson’s *r*	>0.90
Association between SM content and occipital WM β	Normal distribution	Pearson’s *r*	>0.90
Association between SM content and cerebellar WM α	Normal distribution	Pearson’s *r*	>0.90
Association between SM content and cerebellar WM β	Normal distribution	Pearson’s *r*	>0.90
Association between SM content and cerebellar WM γ	Normal distribution	Pearson’s *r*	>0.90
Association between SM content and CC body β	Normal distribution	Pearson’s *r*	>0.90
Association between SM content and CC body γ	Normal distribution	Pearson’s *r*	>0.90
Association between SM content and CC body δ	Normal distribution	Pearson’s *r*	>0.90
Association between SM content and CC genu β	Normal distribution	Pearson’s *r*	>0.90
Association between SM content and CC genu δ	Normal distribution	Pearson’s *r*	>0.90
Association between SM content and CC splenium β	Normal distribution	Pearson’s *r*	>0.90
Association between SM content and CC splenium γ	Normal distribution	Pearson’s *r*	>0.90
Association between SM content and CC splenium δ	Normal distribution	Pearson’s *r*	>0.90

**Table 7.** Statistical table for *in vitro* analyses

		Data structure	Type of test	95% confidence interval	
				CI-2.5%	CI-97.5%
SM 0.5	β-III-tubulin 1	Normal after standardization	*t* test on standardized data	–12.9	18.1
SM 0.5	β-III-tubulin 2	Normal after standardization	*t* test on standardized data	–1.4	21.0
SM 0.5	β-III-tubulin 3	Normal after standardization	*t* test on standardized data	–21.2	13.7
SM 0.5	colocalization 1	Normal after standardization	*t* test on standardized data	–29.9	–6.8
SM 0.5	colocalization 2	Normal after standardization	*t* test on standardized data	–49.2	–36.1
SM 0.5	colocalization 3	Normal after standardization	*t* test on standardized data	–86.5	–70.6
SM 0.5	MBP1	Normal after standardization	*t* test on standardized data	–48.3	–7.8
SM 0.5	MBP2	Normal after standardization	*t* test on standardized data	–64.7	–42.7
SM 0.5	MBP3	Normal after standardization	*t* test on standardized data	–87.3	–72.2
SM 0.5	Myelination1	Normal after standardization	*t* test on standardized data	–29.9	–6.8
SM 0.5	Myelination2	Normal after standardization	*t* test on standardized data	–49.2	–36.1
SM 0.5	Myelination3	Normal after standardization	*t* test on standardized data	–86.5	–70.6
SM 0.5	DAPI1	Normal after standardization	*t* test on standardized data	–11.9	17.8
SM 0.5	DAPI2	Normal after standardization	*t* test on standardized data	–26.3	11.4
SM 0.5	DAPI3	Normal after standardization	*t* test on standardized data	–16.2	13.7
SM 0.5	O4-1	Normal after standardization	*t* test on standardized data	–12.6	18.4
SM 0.5	O4-2	Normal after standardization	*t* test on standardized data	–26.3	11.2
SM 0.5	O4-3	Normal after standardization	*t* test on standardized data	–16.4	14.5
SM 0.5	MBP1	Normal after standardization	*t* test on standardized data	–39.6	–7.7
SM 0.5	MBP2	Normal after standardization	*t* test on standardized data	–37.4	–7.5
SM 0.5	MBP3	Normal after standardization	*t* test on standardized data	–26.6	5.4
SM 5	β-III-tubulin 1	Normal after standardization	*t* test on standardized data	–3.5	8.3
SM 5	β-III-tubulin 2	Normal after standardization	*t* test on standardized data	–17.2	18.0
SM 5	β-III-tubulin 3	Normal after standardization	*t* test on standardized data	–11.5	8.2
SM 5	colocalization 1	Normal after standardization	*t* test on standardized data	–13.7	–4.2
SM 5	colocalization 2	Normal after standardization	*t* test on standardized data	–24.5	–12.2
SM 5	colocalization 3	Normal after standardization	*t* test on standardized data	–69.0	–28.9
SM 5	MBP1	Normal after standardization	*t* test on standardized data	–33.1	–8.0
SM 5	MBP2	Normal after standardization	*t* test on standardized data	–49.8	–32.0
SM 5	MBP3	Normal after standardization	*t* test on standardized data	–72.4	–26.2
SM 5	Myelination1	Normal after standardization	*t* test on standardized data	–13.7	–4.2
SM 5	Myelination2	Normal after standardization	*t* test on standardized data	–24.5	–12.2
SM 5	Myelination3	Normal after standardization	*t* test on standardized data	–69.0	–28.9
SM 5	DAPI1	Normal after standardization	*t* test on standardized data	–2.9	20.5
SM 5	DAPI2	Normal after standardization	*t* test on standardized data	–27.9	28.6
SM 5	DAPI3	Normal after standardization	*t* test on standardized data	–16.2	20.1
SM 5	O4-1	Normal after standardization	*t* test on standardized data	–3.6	18.8
SM 5	O4-2	Normal after standardization	*t* test on standardized data	–28.8	28.5
SM 5	O4-3	Normal after standardization	*t* test on standardized data	–16.0	18.9
SM 5	MBP1	Normal after standardization	*t* test on standardized data	–24.8	5.8
SM 5	MBP2	Normal after standardization	*t* test on standardized data	–49.4	27.3
SM 5	MBP3	Normal after standardization	*t* test on standardized data	–20.2	5.8
SM 25	β-III-tubulin 1	Normal after standardization	*t* test on standardized data	–3.6	27.6
SM 25	β-III-tubulin 2	Normal after standardization	*t* test on standardized data	7.4	20.2
SM 25	β-III-tubulin 3	Normal after standardization	*t* test on standardized data	–19.9	9.0
SM 25	colocalization 1	Normal after standardization	*t* test on standardized data	11.8	21.8
SM 25	colocalization 2	Normal after standardization	*t* test on standardized data	12.2	20.9
SM 25	colocalization 3	Normal after standardization	*t* test on standardized data	56.7	73.1
SM 25	MBP1	Normal after standardization	*t* test on standardized data	–0.1	20.2
SM 25	MBP2	Normal after standardization	*t* test on standardized data	–13.2	5.7
SM 25	MBP3	Normal after standardization	*t* test on standardized data	36.0	70.6
SM 25	Myelination1	Normal after standardization	*t* test on standardized data	11.8	21.8
SM 25	Myelination2	Normal after standardization	*t* test on standardized data	12.2	20.9
SM 25	Myelination3	Normal after standardization	*t* test on standardized data	56.7	73.1
SM 25	DAPI1	Normal after standardization	*t* test on standardized data	9.1	48.6
SM 25	DAPI2	Normal after standardization	*t* test on standardized data	–23.7	31.4
SM 25	DAPI3	Normal after standardization	*t* test on standardized data	0.8	36.4
SM 25	O4-1	Normal after standardization	*t* test on standardized data	9.7	49.4
SM 25	O4-2	Normal after standardization	*t* test on standardized data	–23.3	31.4
SM 25	O4-3	Normal after standardization	*t* test on standardized data	1.7	36.6
SM 25	MBP1	Normal after standardization	*t* test on standardized data	0.4	36.5
SM 25	MBP2	Normal after standardization	*t* test on standardized data	–39.3	24.4
SM 25	MBP3	Normal after standardization	*t* test on standardized data	–16.9	62.0

O4 = olesoxime.

### *In vitro* study

Results obtained from healthy infant observational study shows that dietary SM intake are correlated with increased level of myelination, suggesting that SM may affect the cellular mechanisms linked to myelin formation. to investigate how SM affects myelination, we first investigated how SM affects OPC proliferation, differentiation and maturation in pure OPC cultures, by measuring the total number of cells, O4 positive and MBP positive cells across time, Second, we characterized the effect of SM on axon myelination and axonal growth in an OPC and neurons coculture model by quantifying MBP and β-tubulin III signals alone or their respective colocalization for myelination (for more details, see Materials and Methods).

#### Pure OPC cultures

**Figure 3.**
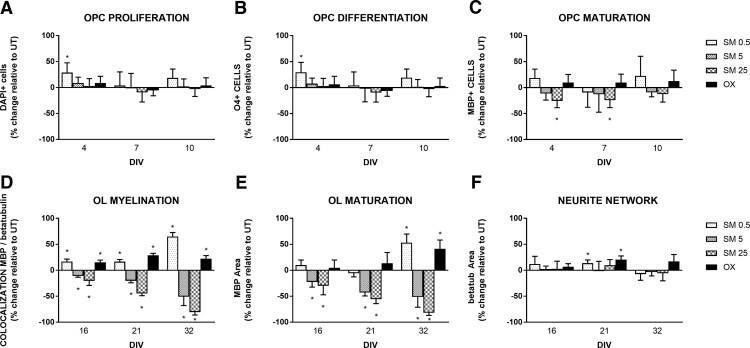
***A–F***, Impact of SM treatment on OPC proliferation, oligodendrocyte maturation, and differentiation in the pure OPC culture and on axon myelination in neuron-oligodendrocyte coculture per treatment group per DIV. Data presented are expressed as percentage change relative to untreated group and represent mean ± SD for 4, 7, and 10 DIV (***A–C***) and for 16, 21, and 32 DIV (***D–F***). Treatment groups are untreated (UT), 0.5 μM SM (SM 0.5), 5 μM SM (SM 5), 25 μM SM (SM 25), and 0.3 μM olesoxime (OX; positive control), *N* = 3 average of minimum two replicates per treatment group per time point. ***A***, OPC proliferation evaluated by total number of cells in percentage change relative to UT. ***B***, Oligodendrocyte differentiation evaluated by total number of O4+ cells in percentage change relative to UT. ***C***, Oligodendrocyte maturation evaluated by total number of MBP+ cells in percentage change relative to UT. ***D***, Myelination in neuron-oligodendrocyte coculture evaluated by total colocalization of MBP and β-III-tubulin signal in percentage change relative to UT. ***E***, MBP immunofluorescence area in percentage change relative to UT. ***F***, β-III-tubulin immunofluorescence area in percentage change relative to UT. Asterisks indicates a significant difference from UT (*p* < 0.022, applying the Benjamini–Hochberg procedure to control the false positive discovery rate at 5% (on 84 performed tests), the threshold for significance was set at *p* < 0.022.

**Figure 4.**
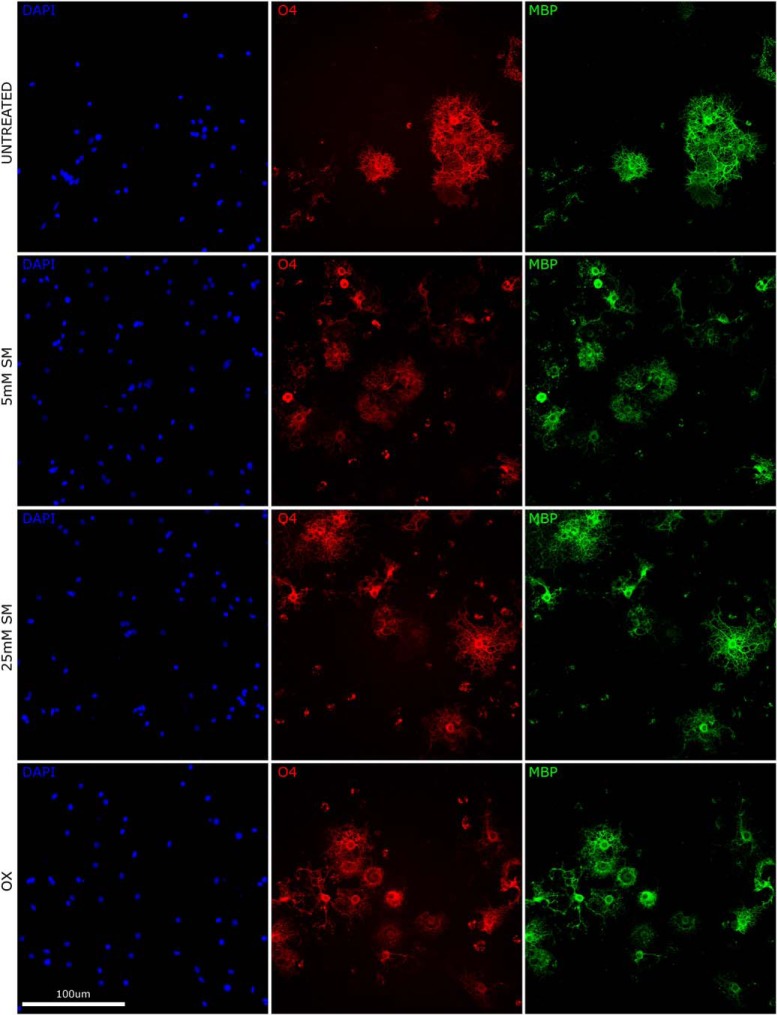
Representative illustration of OPC proliferation, oligodendrocyte maturation, and differentiation in the pure OPC culture per treatment groups at 10 DIV. Co-immunostaining with DAPI (blue), anti-O4 (red), and anti-MBP (green) of coculture at 21 DIV for the untreated (UT), 0.5 μM SM (SM 0.5), 5 μM SM (SM 5), 25 μM SM (SM 25), and 0.3 μM olesoxime (OX; positive control) groups. Proliferation was measured by number of cells stained with DAPI, maturation by number of O4+ cells, and differentiation by number of MBP+ cells.

We observed 0.5 µM SM resulted in increased total cell number, significantly at 4 DIV (effect size: 29.6%, *p* = 0.0133; Fig. 3*A*). SM at higher doses (5 and 25 µM) had no impact on total cell number (*p* > 0.1; Fig. 3*A*). Cell differentiation (O4+ cells) was significantly increased by 0.5 µM SM at 4 DIV (effect size 28.8%; *p* = 0.0133; Fig. 3*B*). SM at higher doses (5 and 25 µM) had no impact on cell differentiation (*p* > 0.1; Fig. 3*B*). 0.5 and 5 µM SM showed no impact on cell maturation (MBP+ cells, *p* > 0.1; Fig. 3*C*); 25 µM SM resulted in a decrease of cell maturation, significantly at 4 and 7 DIV (effect size: –23.6, –22.5%; *p* = 0.0126 and *p* = 0.0103, respectively; Fig. 3*C*). Olesoxime treatment had no effect on total cell numbers, cell differentiation or maturation (*p* > 0.1; Fig. 3*A–C*). Overall, these results show that SM treatment at 0.5 µM resulted in increased OPC proliferation and differentiation (for representative images, see Fig. 4). With increased SM concentration, the effect gradually shifts toward a negative impact on differentiation and no impact on proliferation and maturation.

#### Co-cultures

**Figure 5.**
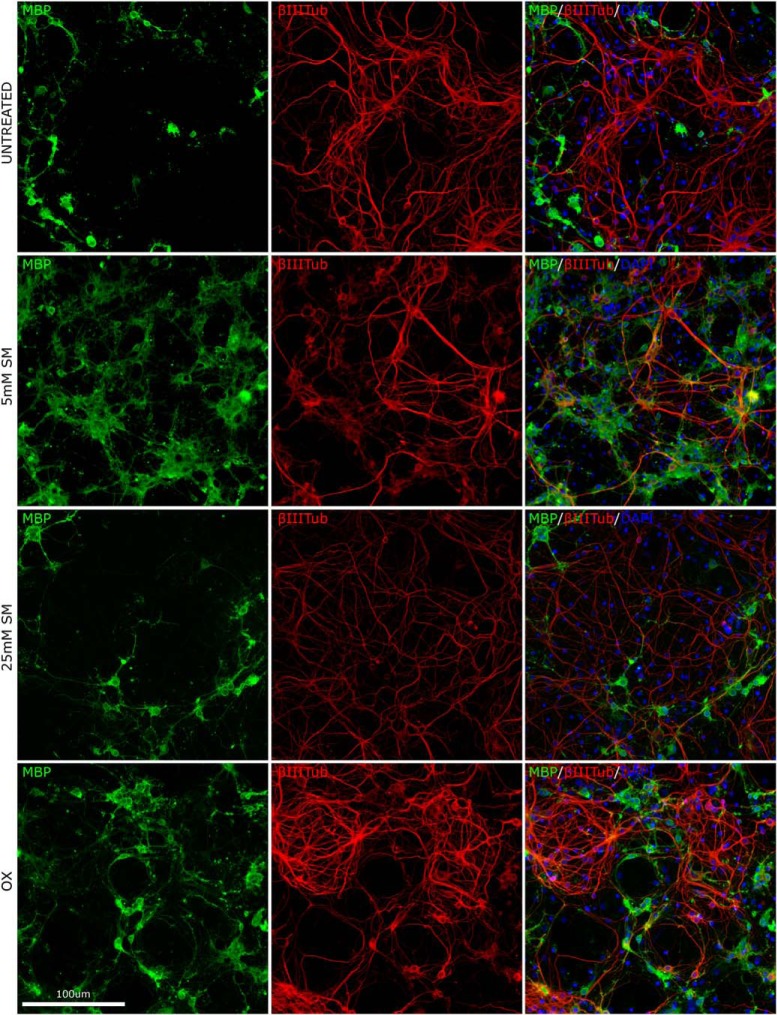
Representative illustration of neuron-oligodendrocyte coculture at 32 DIV. Coimmunostaining with MPB (green), anti-β-III-tubulin (red), and DAPI (blue) of coculture at 32 DIV for the untreated (UT), 0.5 μM SM (SM 0.5), 5 μM SM (SM 5), 25 μM SM (SM 25), and 0.3 μM olesoxime (OX; positive control) groups. Myelination was quantified by the overlap of MBP (green) and β-III-tubulin (red) staining.

We observed 0.5 µM SM significantly increased axon myelination (co-localization of MBP and β-III-tubulin markers) at 16, 21, and 32 DIV (effect size: 16.8%, 16.5%, 64.9%; *p* = 0.0003, *p* = 0.0002, and *p* = 0.0001, respectively; Fig. 3*D*, and see representative images in Fig. 5). On the contrary, SM treatment at higher doses significantly reduced myelination at 16, 21, and 32 DIV for 5 µM (effect size: –9.0%, –18.3%, –48.9%; *p* = 0.0047, *p* = 0.0006, and *p* = 0.0015, respectively; Fig. 3*D*) and for 25 µM (effect size: –18.3%, –42.7%, –78.6%; *p* < 0.0095, *p* < 0.0001, and *p* < 0.0001, respectively; Fig. 3*D*). Treatment at 0.5 µM SM significantly increased myelinating oligodendrocytes maturation (total MBP area) at 32 DIV (effect size: 53.3%; *p* = 0.0005; Fig. 3*E*). Similarly to the effect observed on myelination, higher doses of SM resulted in a significant reduction of MBP signal at 16, 21, and 32 DIV; for 5 µM (effect size: –20.5%, –40.9%, –49.3%; *p* = 0.0084, *p* = 0.0001, and *p* = 0.0027, respectively; Fig. 3*E*); and 25 µM (effect size: –28.0%, –53.7%, –79.7%; *p* = 0.0163, *p* = 0.0001, and *p* = 0.0001, respectively; Fig. 3*E*); 0.5 µM SM increased axonal growth (β-III-tubulin) significantly at 21 DIV (effect size: 13.8%, *p* = 0.0026; Fig. 3*F*). Olesoxime significantly increased myelination at 16, 21, and 32 DIV (effect size: 15.1%, 28.7%, 22.2%; *p* = 0.0004, *p* = 0.0001, and *p* = 0.0003, respectively; Fig. 3*D*) and axonal growth (β-III-tubulin) at 21 DIV (effect size: 20.5%; *p* = 0.0008; Fig. 4*F*); and increased significantly myelinating oligodendrocyte (total MBP signal) at 32 DIV (effect size: 41.2%, *p* = 0.0019; Fig. 3*D*). These results suggest that SM at concentration similar to that found in human CSF is capable of enhancing axon myelination and to some extent axonal growth in an *in vitro* coculture model of myelination (for representative images, see Fig. 5). With increased SM concentration, the effect gradually shifts toward a negative impact on myelination.

## Discussion

To our knowledge, this is the first study in term-born neurotypically developing children that links dietary SM intake from infant nutrition products to cognitive development and brain myelination. Although preliminary, those findings provide important knowledge building and should be followed by efficacy studies. Our human observational data suggests that SM levels in infant nutrition products (28–71 mg/l) are positively correlated with changes in myelination in specific brain regions in the first two years of life. In addition, SM levels were also positively correlated with cognitive performance within normal ranges of development (ELC range = 91.5–104.2). We found verbal functioning (e.g., auditory comprehension and memory, speaking ability and language formation; Dumont et al., 2000) to be more sensitive to early life nutrition than non-verbal functioning (e.g., visual processing and visual memory, motor control and coordination skills; Dumont et al., 2000).

These association findings in typically developing children may complement the preliminary findings of Tanaka et al. (2013) in low birth weight infants. In this study, the authors reported an improvement of cognitive and behavioral outcomes at 12 and/or 18 months of age following a SM-fortified infant formula intervention in the first two months of life. Infants in that study were mostly mixed-fed (formula in addition to breast milk), thus effects cannot be attributed to the SM-fortified infant formula alone. Although exploratory, both studies may support the role of dietary SM in cognitive development from 12–24 months of age. Before any impact on cognitive development, our findings suggest a modulation of myelin development in the brain, possibly via the involvement of SM in the structure and function of myelin (Ledesma et al., 1999; Don et al., 2014). Higher levels of SM in the first three months of life were associated (*p* < 0.05 FDR) with higher levels of brain myelin content in the second year of life, particularly in the cerebellum, occipital lobe, visual cortex, internal capsule, parietal lobe, and motor cortices. Furthermore, our findings indicate an impact of SM on the pattern of developmental myelination in favor of a later onset as well as a more prolonged rate of myelination. This seems particularly relevant in the context of development, as dysregulations and deviations from the developmental pattern, rather than overall myelin quantity, may contribute to the production of human psychiatric disturbances, such as autism spectrum disorders, learning deficits, sensory processing delay disorder, attention deficit hyperactivity disorder, and Retts syndrome (Herbert et al., 2004; Bartzokis 2005; Bercury and Macklin, 2015).

Our supporting *in vitro* studies show that physiologic levels of SM positively affect the proliferation, differentiation and myelination capacity of oligodendrocyte while higher concentrations may have a negative impact. However, it is unlikely that SM itself is transported to the brain and incorporated into myelin sheath directly. SM is metabolized in the small intestine into ceramide and sphingosine by alkaline sphingomyelinase (SMase; Nyberg et al., 1997; Ohlsson et al., 2010). Additional studies show that sphingosine and ceramide derived from sphingolipid hydrolysis are rapidly taken up by intestinal cells, then either degraded into fatty acids or reincorporated into complex sphingolipids that could be associated with lipoprotein particles including chylomicron, very low-density lipoprotein (VLDL), LDL, and high-density lipoprotein (HDL; Nilsson and Duan, 2006). SM has been shown to be the major sphingolipid in LDL and HDL (Vesper et al., 1999). Injection of HDL containing radiolabeled SM suggest some direct uptake of SM to the brain, however the majority was resynthesized in the brain (Bentejac et al., 1988, 1989). Since the brain appears to be capable of *de novo* synthesis of sphingolipids, SM and its constituents could be used for *de novo* synthesis of main myelin lipids such as cerebrosides to support myelin formation during brain development. However, the time course of intact *de novo* synthesis of sphingolipids in newborns is not yet well understood. It is likely that infants depend on external sources in their first weeks of life. This hypothesis is supported by the greater uptake of radiolabeled SM injected in blood observed in 20-d-old rats compare to adult rats (Bentejac et al., 1989). Additional experiments are necessary to fully characterize how orally ingested SM contribute to myelinogenesis, especially at different stages of infant and child development.

In our *in vitro* experiments, we used SM in a liposome form to facilitate its cellular uptake. We observed that the lowest concentration of SM, corresponding to the one found in human CSF, promotes OPC proliferation and differentiation in pure OPC cultures and enhances myelination in coculture. Higher concentrations of SM have no effect on OPC proliferation and OPC maturation or negatively affect OPC differentiation, OL myelination, and OL maturation. While the low SM concentrations transiently enhanced dendritic arborization in coculture, higher concentrations failed to yield an effect. Our results suggest that SM may have different effects on oligodendrocyte and neuron physiology that may be linked to its role in multiple cell processes. Additional experiments will be needed to characterize the exact mechanism of SM in myelination and axonal growth.

Limitations of our study include the retrospective analyses of the infant nutrition products. While product and feeding information was acquired during the study period, product samples were acquired at the study end and SM analyses were performed retrospectively using a single time assay. Thus, the nutritional formulations of the reported products may have changed in the years from the earliest imaging data up to the time of our nutritional analysis. Furthermore, we observed a temporal delay between infant feeding and impact on myelination and cognitive development. It is likely that follow-on complementary feeding and nutrition, as well as other environmental influences previously associated with cognitive development and outcomes in children, such as parent-child interaction (Swain et al., 2007), physical activity levels (Best, 2010), and sleep duration and quality (Peirano and Algarín, 2007) are unexamined contributors to our results. Another important caveat to our study is the absence of bioavailability information in both our observational as well as our *in vitro* data. As discussed above, SM is metabolized and resynthesized and relevant amounts are unlikely to reach the brain directly (Nyberg et al., 1997). Observed associations may therefore be the result of SM metabolites influencing brain and cognitive development rather than SM itself. Indeed, more studies are needed to understand bioavailability, brain uptake and mechanisms of action of dietary SM and its metabolites. The relatively small sample size per sub-group of infant nutrition product-fed infants (*N* = 21, *N* = 28, and *N* = 39, respectively) should be considered when interpreting the findings of the study. Although not unusual for pediatric neuroimaging studies, the sample sizes are low. At this stage, we consider our findings preliminary and in support of the need to further investigate the role of dietary SM in normal brain and cognitive development.

In conclusion, our first findings from a clinical observational study indicate an impact of dietary SM on cognitive development in healthy term-born children. This effect may be mediated through oligodendrocytes proliferation and differentiation as supported by our *in vitro* experiments. Randomized controlled trials, ideally of longitudinal nature to best reflect development and maturation, are needed to substantiate efficacy for dietary SM related cognitive benefits. In addition, complementary preclinical studies on bioavailability and brain uptake of SM and its metabolites are needed. Furthermore, SM doses for oral intake and efficacy need to be identified to provide appropriate recommendations for infant and child nutrition.

## Synthesis

Reviewing Editor: Robert Kalb, Northwestern University Decisions are customarily a result of the Reviewing Editor and the peer reviewers coming together and discussing their recommendations until a consensus is reached. When revisions are invited, a fact-based synthesis statement explaining their decision and outlining what is needed to prepare a revision will be listed below. The following reviewer(s) agreed to reveal their identity: Amy Waldman.

The emendations are fine but Reviewer #1 finds that s/he is “confused about what they found with respect to correlation with myelination in the humans”. Please clarify.

## Author Response

June 2019

Robert Kalb

Reviewing Editor,

eNeuro

Dear Dr. Kalb,

Re: Decision on eNeuro eN-NWR-0421-18R1

Thank you again for considering our original research article for publication in eNeuro and for allowing us to revise and resubmit the manuscript. We value the feedback provided by the reviewers. Please see below for our response to the one remaining comment.

Yours sincerely,

Barry O'Neill

Corresponding author

---------------------------------------------

Synthesis of Reviews:

Significance Statement Comments for Author (Required):

A nicely done study relating dietary sphingomyelin and brain development

Comments on the Visual Abstract for Author (Required):

N/A

Synthesis Statement for Author (Required):

The emendations are fine but Reviewer #1 finds that s/he is “confused about what they found with respect to correlation with myelination in the humans”. Please clarify.

Response:

Based on a lack of data as regards benefits of dietary sphingomyelin on brain & cognitive development, the paper summarizes preliminary data establishing a link between dietary sphingomyelin and cognition through myelination.

The main findings with respect to myelination in healthy infants were:

1. Cross sectional findings: a correlation between sphingomyelin levels present in the infant nutrition products and brain myelin water fraction in multiple brain regions at 12-24 months of age (but not at 1-12 months of age) (see figure 1). Specifically, higher levels of sphingomyelin in the products were correlated with higher levels of myelin in these brain regions.

2. Longitudinal findings: we saw that early life SM content is significantly correlated to brain myelin development profiles throughout the brain (Fig. 2 and Table 5) and, in particular with respect to the initial onset of myelination (Gompertz curve parameter β), i.e. greater SM content being associated with a later myelin onset, and initial growth rate (Gompertz curve parameter γ), i.e. greater SM content being associated with a slower but more prolonged rate of myelination.

The authors believe we have made these points clearly and they are adequately presented in the body of the manuscript, as well as the figures, figure legends and tables. If the reviewer feels the manuscript would benefit from further explanation of these points in specific areas, we would appreciate guidance on where they would like to see us elaborate further.
